# Phase 1b/2 trial of tepotinib in sorafenib pretreated advanced hepatocellular carcinoma with MET overexpression

**DOI:** 10.1038/s41416-021-01334-9

**Published:** 2021-04-06

**Authors:** Thomas Decaens, Carlo Barone, Eric Assenat, Martin Wermke, Angelica Fasolo, Philippe Merle, Jean-Frédéric Blanc, Véronique Grando, Angelo Iacobellis, Erica Villa, Joerg Trojan, Josef Straub, Rolf Bruns, Karin Berghoff, Juergen Scheele, Eric Raymond, Sandrine Faivre

**Affiliations:** 1 University Grenoble Alpes, Department of Hepato-Gastroenterology and Digestive Oncology, CHU Grenoble Alpes, Institute for Advanced Biosciences INSERM U1209, Grenoble, France; 2 Medical Oncology, Policlinico Universitario A. Gemelli, Roma, Italy; 3 Medical Oncology, CHU Saint Eloi, Montpellier, France; 4 NCT/UCC Early Clinical Trial Unit, University Hospital Carl-Gustav-Carus, Dresden, Germany; 5 Oncology, IRCCS San Raffaele, Milan, Italy; 6 Service d’Hépato-Gastro-Entérologie, Hôpital de la Croix Rousse, Lyon, France; 7 Service d’Hépato-Gastroentérologie et d’Oncologie Digestive, Groupe Hospitalier Haut-Lévêque, CHU Bordeaux, Pessac, France; 8 Service Hépatologie, Hôpital Jean-Verdier, Bondy, France; 9 Reparto di Gastroenterologia ed Endoscopia Digestiva, Ospedale Casa Sollievo della Sofferenza IRCCS, San Giovanni Rotondo, Italy; 10 Division of Gastroenterology Policlinico di Modena, Modena, Italy; 11 Gastrointestinal Oncology, Goethe University Hospital, Frankfurt, Germany; 12 Clinical Biomarker & Companion Diagnostics, Merck KGaA, Darmstadt, Germany; 13 Biostatistics, Merck KGaA, Darmstadt, Germany; 14 Global Patient Safety Innovation, Merck KGaA, Darmstadt, Germany; 15 Global Clinical Development Oncology, Merck KGaA, Darmstadt, Germany; 16 Medical Oncology, Paris-St Joseph Hospital, Paris, France; 17 Medical Oncology, Saint-Louis Hospital & Paris 7 University, Paris, France

**Keywords:** Hepatocellular carcinoma, Molecularly targeted therapy

## Abstract

**Background:**

This Phase 1b/2 study evaluated tepotinib, a highly selective MET inhibitor, in US/European patients with sorafenib pretreated advanced hepatocellular carcinoma (aHCC) with MET overexpression.

**Methods:**

Eligible adults had aHCC, progression after ≥4 weeks of sorafenib, and, for Phase 2 only, MET overexpression. Tepotinib was administered once daily at 300 or 500 mg in Phase 1b (‘3 + 3’ design), and at the recommended Phase 2 dose (RP2D) in Phase 2. Primary endpoints were dose-liming toxicities (DLTs; Phase 1b) and 12-week investigator-assessed progression-free survival (PFS; Phase 2).

**Results:**

In Phase 1b (*n* = 17), no DLTs occurred and the RP2D was confirmed as 500 mg. In Phase 2 (*n* = 49), the primary endpoint was met: 12-week PFS was 63.3% (90% CI: 50.5–74.7), which was significantly greater than the predefined null hypothesis of ≤15% (one-sided binomial exact test: *P* < 0.0001). Median time to progression was 4 months. In Phase 2, 28.6% of patients had treatment-related Grade ≥3 adverse events, including peripheral oedema and lipase increase (both 6.1%).

**Conclusions:**

Tepotinib was generally well tolerated and the RP2D (500 mg) showed promising efficacy and, therefore, a positive benefit–risk balance in sorafenib pretreated aHCC with MET overexpression.

**Trial Registration:**

ClinicalTrials.gov: NCT02115373.

## Background

Liver cancer is the third leading cause of cancer-related death worldwide, with more than 780,000 deaths due to the disease each year.^1^ The incidence of hepatocellular carcinoma (HCC), which accounts for 90% of primary liver cancers,^2^ is still rising, in part driven by high rates of chronic viral hepatitis and increasing obesity-related liver disease.^3^ HCC detection remains late, and only ~30–40% of US/European patients have curative treatment options at the time of diagnosis.^4^ Many have poor clinical conditions and an unfavourable prognosis due to underlying liver dysfunction and/or advanced disease stage, i.e. Barcelona Clinic Liver Cancer stage C.^4,5^ There is an urgent unmet need for new therapies and combinations that can improve patient outcomes in this setting.

Until recently, sorafenib was the only approved systemic targeted therapy for advanced HCC (aHCC).^6^ As first-line therapy, sorafenib provides a modest but significant improvement in overall survival (OS), but its activity is almost universally hampered by the development of drug resistance.^7,8^ The options for aHCC treatment have since expanded: lenvatinib and atezolizumab + bevacizumab are approved for first-line treatment and regorafenib, ramucirumab (in patients with alpha-fetoprotein [AFP] elevation) and cabozantinib are approved for use post-sorafenib in both the US and Europe.^9–13^ In the US, approvals have also been granted for pembrolizumab and nivolumab ± ipilimumab in the second-line setting.^14–17^ Despite the availability of novel therapies, the overall prognosis for aHCC is poor.^18^ Furthermore, dependent on the results of ongoing trials and drug approvals, greater use of immunotherapies for upfront therapy may lead to an increased need for effective treatment options for subsequent lines.^6^

MET, the tyrosine kinase receptor for hepatocyte growth factor (HGF), is often dysregulated in HCC, which can promote rapid tumour growth and aggressive invasiveness.^18^ Approximately 50% of HCCs are reported to harbour *MET* alterations, including gene mutation in 4%, gene amplification in 24% and overexpression of mRNA and protein in 50% and 28%, respectively.^18^ Moreover, MET expression has been reported to increase following treatment with sorafenib,^19^ suggesting that MET overexpression may contribute to sorafenib resistance. Aberrant MET activation has been implicated in resistance to vascular endothelial growth factor receptor (VEGFR) inhibitors in a number of solid tumours.^20–22^ Following sorafenib treatment, MET overexpression is associated with a poor prognosis, with significantly shorter median OS compared with aHCC without MET overexpression.^23^ MET is, therefore, a therapeutic target in HCC after failure of sorafenib,^18^ and probably also after other treatments that target the VEGF pathway.

Tepotinib is an orally available, potent and highly selective MET inhibitor that has shown pronounced anti-tumour activity in MET-dependent preclinical models in vivo, in patients with a range of solid tumours (including HCC), and in patients with advanced non-small cell lung cancer (NSCLC) with *MET* exon 14 skipping.^24–26^ In combination with gefitinib, tepotinib improved efficacy compared with chemotherapy in patients with advanced, epidermal growth factor receptor (EGFR)-mutant NSCLC with MET overexpression or *MET* amplification and acquired resistance to EGFR inhibitor therapy in a randomised Phase 1b/2 trial^27^ and a Phase 2 study of tepotinib plus osimertinib in patients with acquired resistance to first-line osimertinib due to *MET* amplification in an ongoing trial (INSIGHT 2; NCT03940703). Tepotinib in combination with cetuximab is also being investigated in patients with *RAS/BRAF* wild-type left-sided metastatic colorectal cancer and acquired resistance to anti-EGFR antibody-targeted therapy due to *MET* amplification in a Phase 2 study (NCT04515394).

In HCC, tepotinib has demonstrated preclinical activity in patient-derived primary liver cancer explants, in which sensitivity to the drug was associated with MET overexpression.^26^ These data, together with the rationale for targeting MET in HCC, led to the design of two Phase 1b/2 trials of tepotinib in patients with aHCC with MET overexpression. The trials incorporated Phase 1b parts to establish the recommended Phase 2 dose (RP2D) of tepotinib in patients with aHCC and Child–Pugh Class A liver function, who were excluded from the first-in-human Phase 1 trial.^24^ One of the Phase 1b/2 trials in aHCC with MET overexpression was conducted in Asian patients without prior systemic anti-cancer therapy and demonstrated a significant increase in time to progression (TTP) with tepotinib versus sorafenib.^28^ Here, we report the second aHCC Phase 1b/2 study, which investigated tepotinib in Western patients with MET overexpression who had previously failed treatment with sorafenib (NCT02115373).

## Methods

### Study design and objectives

This was an open-label, multicentre, integrated, Phase 1b/2 trial conducted across Europe and the US in patients with aHCC who had failed sorafenib treatment. Phase 1b was an open-label, single-arm, dose-escalation study with a classic ‘3 + 3’ design, and the primary objective was to confirm the RP2D of tepotinib. Phase 2 was an open-label, single-arm, non-randomised study to evaluate the activity of tepotinib in patients with MET overexpression (Supplementary Fig. 1).

All patients provided written informed consent for participation in the study. The study was done in accordance with the Declaration of Helsinki, International Council for Harmonisation guidelines for Good Clinical Practice, local laws and applicable regulatory requirements. The study was approved by the institutional review board or independent ethics committee of each centre.

### Patients

In both study phases, eligible patients were ≥18 years old with aHCC, Child–Pugh Class A liver function score, Eastern Cooperative Oncology Group performance status 0 or 1, and measurable disease defined by Response Evaluation Criteria In Solid Tumors (RECIST) v1.1. Patients were also required to have ≥4 weeks of prior sorafenib treatment, which was discontinued ≥2 weeks before tepotinib initiation due to either intolerance or disease progression. Exclusion criteria included any prior systemic anti-cancer treatment for aHCC (besides sorafenib) and presence of symptomatic or untreated brain metastases. Full inclusion and exclusion criteria are shown in Supplementary Table 1.

In Phase 2, patients were required to have MET overexpression, based on assessment of a tumour biopsy sample collected after sorafenib discontinuation and within 28 days before the first day of study treatment. MET overexpression was defined as moderate (2+) or strong (3+) staining for MET in ≥50% of tumour cells by immunohistochemistry (IHC) using the pharmDx anti-total MET (D1C2) rabbit monoclonal antibody (Dako, Agilent Technologies, Inc., Santa Clara, CA) (Supplementary Table 2). MET status was not required for Phase 1b, but was determined retrospectively in a biopsy sample collected within 28 days before the first day of study treatment. *MET* amplification status was determined for patients in Phase 1b and 2 using formalin-fixed paraffin-embedded tumour material by fluorescent in situ hybridisation. *MET* amplification was defined as *MET*:*CEP7* ratio ≥2 or mean gene copy number ≥5.

### Treatment administration

In Phase 1b, patients received oral tepotinib hydrochloride hydrate in continuous 21-day cycles at one of two daily dose levels: 300 and 500 mg (containing 270 and 450 mg, respectively, of the active moiety in free base form), each given once daily (QD). Additional patients were enrolled at the RP2D. All patients were treated QD with the RP2D of tepotinib in the Phase 2 part of the study. Tepotinib was administered until disease progression, intolerable toxicity, death or withdrawal from treatment.

### Study endpoints and assessments

The primary endpoint in Phase 1b was incidence of dose-limiting toxicities (DLTs) during Cycle 1. Details of DLTs are defined in Supplementary Table 3. Pharmacokinetics (PK) endpoints, including C_max_, and area under the concentration–time curve over the dosing interval at steady state (AUC_τ,ss_), were evaluated at Cycle 1, Day 15 as a secondary endpoint in Phase 1b. Selected efficacy measures were evaluated as secondary endpoints in Phase 1b.

In Phase 2, the primary endpoint was progression-free survival (PFS) status after 12 weeks of treatment, as assessed by the investigator per RECIST v1.1. Secondary endpoints included TTP, time to symptomatic progression, objective response rate (ORR), disease control rate (DCR) (all investigator-assessed and per RECIST v1.1), PFS per modified RECIST v1.1 (investigator-assessed), OS and biological response (defined as a decrease in AFP >20%). As sensitivity analyses, PFS and TTP were also evaluated by independent review. Safety was assessed via reporting of adverse events (AEs), graded according to National Cancer Institute Common Terminology and Criteria for Adverse Events v4.0.

In both phases, tumour assessments were performed per RECIST v1.1 and modified RECIST^29^ v1.1 on Day 1 of every second cycle from Cycle 3 to 13, and Day 1 of every fourth cycle thereafter. Exploratory endpoints included biomarkers of MET pathway activity (including *MET* amplification status).

### Statistical analysis

Continuous variables, including PK data, were summarised using descriptive statistics; qualitative variables were summarised by means of counts and percentages. In both phases, efficacy endpoints and AEs were analysed in the intention-to-treat/safety analysis set (SAS), which includes all patients who were administered ≥1 dose of tepotinib.

In Phase 1b, DLTs were assessed during Cycle 1 in all patients who received ≥80% of the planned cumulative dose of tepotinib and patients who stopped treatment due to a DLT. Patients who were replaced during Cycle 1 were excluded from the analysis set.

For the Phase 2 primary endpoint, the null hypothesis that the 12-week progression-free rate is ≤15% (based on historical data)^23^ was tested against a one-sided alternative hypothesis that the 12-week progression-free rate is 30%, using a binomial exact test with an α value of 0.05. A sample size of 48 patients was required to achieve a power of at least 80%.

Tumour response (best overall response, ORR, DCR) and biological response were summarised, along with corresponding two-sided exact Clopper–Pearson 90% confidence intervals (CIs). Kaplan–Meier estimates of median PFS, TTP, time to symptomatic progression and OS are presented, along with 90% CIs (calculated using the two-sided exact Clopper–Pearson method). All statistical analyses were performed using SAS^®^ Software version 9.4 (Statistical Analysis System, SAS-Institute, Cary, North Carolina, US).

## Results

### Patients and treatment

Patients were enrolled between May 2014 and February 2017. In Phase 1b, 24 patients were screened and 17 received treatment: four patients in the 300 mg cohort and 13 patients in the 500 mg cohort (Supplementary Fig. 2a). In Phase 2, 155 patients were screened and 49 were treated with tepotinib 500 mg, forming the intention-to-treat/SAS (Supplementary Fig. 2b). Baseline characteristics of patients in Phase 1b and 2 were representative of the target population with aHCC (Table 1). The median duration of treatment with tepotinib was 2.7 months (range, 0–23 months) in Phase 1b and 3.02 months (range, 0.03–16.49 months) in Phase 2.

**Table 1:** Patient demographics and baseline characteristics (Phase 1b and 2).

	Phase 1b			Phase 2
	300 mg/day	500 mg/day	Total	500 mg/day
	(*n* = 4)	(*n* = 13)	(*N* = 17)	(*N* = 49)
Age, years, median (range)	65.5 (58−73)	69.0 (51−79)	69.0 (51−79)	66.0 (19−82)
<65 years, *n* (%)	1 (25.0)	3 (23.1)	4 (23.5)	21 (42.9)
≥65 years, *n* (%)	3 (75.0)	10 (76.9)	13 (76.4)	28 (57.1)
Sex, *n* (%)				
Male	2 (50.0)	11 (84.6)	13 (76.5)	41 (83.7)
Female	2 (50.0)	2 (15.4)	4 (23.5)	8 (16.3)
Race, *n* (%)				
White	1 (25.0)	8 (61.5)	9 (52.9)	26 (53.1)
Black	0 (0.0)	0 (0.0)	0 (0.0)	1 (2.0)
Asian	0 (0.0)	0 (0.0)	0 (0.0)	2 (4.1)
Other	0 (0.0)	1 (7.7)	1 (5.9)	0 (0.0)
Missing	3 (75.0)	4 (30.8)	7 (41.2)	20 (40.8)
ECOG PS at baseline, *n* (%)				
0	3 (75.0)	10 (76.9)	13 (76.5)	25 (51.0)
1	1 (25.0)	3 (23.1)	4 (23.5)	24 (49.0)
Cause of HCC (investigator-assessed), *n* (%)				
HBV				4 (8.2)
HCV				13 (26.5)
Alcohol-induced cirrhosis				11 (22.4)
Non-alcoholic steatohepatitis				2 (4.1)
Steatosis				2 (4.1)
Other^a^				16 (32.7)
Missing				1 (2.0)
Vascular invasion, *n* (%)				
Yes	1 (25.0)	5 (38.5)	6 (35.3)	12 (24.5)
No	0 (0.0)	5 (38.5)	5 (29.4)	24 (49.0)
Missing	3 (75.0)	3 (23.1)	6 (35.3)	13 (26.5)
MET IHC, *n* (%)^b^				
IHC 0	0 (0.0)	1 (7.7)	1 (5.9)	
IHC 1+	1 (25.0)	4 (30.8)	5 (29.4)	
IHC 2+	3 (75.0)	4 (30.8)	7 (41.2)	41 (83.7)
IHC 3+	0 (0.0)	2 (15.4)	2 (11.8)	8 (16.3)
Missing	0 (0.0)	2 (15.4)	2 (11.8)	0 (0.0)
*MET* amplification, *n* (%)^c^				
Present	0 (0.0)	0 (0.0)	0 (0.0)	6 (12.2)
Absent	4 (100.0)	10 (76.9)	14 (82.4)	43 (87.8)
Missing	0 (0.0)	3 (23.1)	3 (17.6)	0 (0.0)
AFP elevation, *n* (%)				
<200 µg/L	1 (25.0)	9 (69.2)	10 (58.8)	20 (40.8)
≥200 µg/L	3 (75.0)	3 (23.1)	6 (35.3)	27 (55.1)
Missing	0 (0.0)	1 (7.7)	1 (5.9)	2 (4.1)

Please note, as numbers have been rounded to one decimal place, some columns may not total 100%.

*AFP* alpha-fetoprotein, *ECOG PS* Eastern Cooperative Oncology Group performance status, *HBV* hepatitis B virus, *HCC* hepatocellular carcinoma, *HCV* Hepatitis C virus, *IHC* immunohistochemistry.

^a^Other investigator-assessed root causes of HCC were: metabolic cirrhosis (*n* = 1), adenomatous hyperplasia (*n* = 1), hemochromatosis (*n* = 1), regenerative nodular hyperplasia (*n* = 1), previous drug abuse (*n* = 1); cirrhosis (*n* = 1); both alcohol-induced cirrhosis and HCV (*n* = 1) and idiopathic/unknown/none/not assessable (*n* = 9).

^b^In Phase 1b, patients were not required to have MET overexpression.

^c^*MET* amplification is defined as *MET:CEP7* ratio ≥2 or mean gene copy number ≥5.

### Safety

In Phase 1b, no DLTs occurred in either dose cohort, and the RP2D was, therefore, confirmed as 500 mg QD. Of 17 patients, 16 (94.1%) experienced an AE of any cause in Phase 1b, of which ten (58.8%) experienced Grade ≥3 events of any cause.

In Phase 2, 48 of 49 patients (98.0%) experienced an AE of any cause; 28 (57.1%) experienced Grade ≥3 AEs of any cause. The most common AEs of any cause in Phase 2 were peripheral oedema (65.3%), ascites (34.7%) and diarrhoea (32.7%). Peripheral oedema was also the most common AE of any cause in Phase 1b (76.5%).

Three of 17 patients (17.6%) in Phase 1b and 17 of 49 (34.7%) in Phase 2 permanently discontinued treatment due to AEs of any cause. These AEs were considered to be treatment-related for one patient (5.9%) in Phase 1b (peripheral oedema) and eight patients (16.3%) in Phase 2 (peripheral oedema [*n* = 5], ascites [*n* = 1], aspartate aminotransferase increased [*n* = 1], blood creatinine increased [*n* = 1] and hypoglycaemic coma [*n* = 1]). Four patients (23.5%) in Phase 1b and eight (16.3%) in Phase 2 experienced AEs, of any cause, that led to death. In one instance, this was deemed by the investigator to be related to study treatment (hypoglycaemic coma, patient in Phase 2). This patient had type 1 diabetes with a history of multiple insulin medications to manage the condition.

In Phase 2, AEs related to study treatment were reported in 41 of 49 patients (83.7%); these were Grade ≥3 in 14 patients (28.6%) (Table 2). The most common treatment-related AEs were peripheral oedema (38.8%), asthenia (22.4%), fatigue (18.4%), diarrhoea (16.3%) and nausea (14.3%). The most common Grade ≥3 treatment-related AEs were peripheral oedema (6.1%) and increased lipase (6.1%). The incidence of treatment-related AEs was similar in Phase 1b, with 82.4% of patients experiencing at least one event, and 23.5% experiencing at least one Grade ≥3 event.

**Table 2:** Phase 2: Treatment-emergent adverse events of any grade and Grade ≥3.

Event	Tepotinib	
	Any grade	Grade ≥3
≥1 adverse event of any cause,^a^ *n* (%)	48 (98.0)	28 (57.1)
≥1 treatment-related adverse event, *n* (%)	41 (83.7)	14 (28.6)
Treatment-related adverse event in ≥5% of patients, *n* (%)		
Peripheral oedema	19 (38.8)	3 (6.1)
Asthenia	11 (22.4)	0
Fatigue	9 (18.4)	0
Diarrhoea	8 (16.3)	0
Nausea	7 (14.3)	0
Ascites	6 (12.2)	2 (4.1)
Hypoalbuminaemia	5 (10.2)	0
Decreased appetite	4 (8.2)	0
Vomiting	4 (8.2)	0
Blood creatinine increased	3 (6.1)	1 (2.0)
Lipase increased	3 (6.1)	3 (6.1)
Pruritus	3 (6.1)	0

^a^Treatment-related adverse events are defined as events that occur within the day of first dose of trial treatment, up until 33 days after last dose of treatment.

### Efficacy

In Phase 1b, best overall response was partial response (PR) in two of 17 patients (11.8%), both treated with 300 mg tepotinib (Table 3). Objective responses were seen in two of four patients (50%) in the 300 mg cohort and no patients (of 13; 0%) in the 500 mg cohort. Disease control was seen in two of four patients (50%) in the 300 mg cohort and four of 13 (30.8%) in the 500 mg cohort; six of 17 (35.3%) overall. Objective responses were seen in no patients (*n* = 1; 0%) with MET IHC 0, one patient (*n* = 5; 20%) with MET IHC 1+, one patient (*n* = 7; 14.3%) with MET IHC 2+ and no patients (*n* = 2; 0.0%) with MET IHC 3+. The DCR was 100.0% for patients with MET IHC 0, 40.0% for MET IHC 1+, 42.9% for MET IHC 2+ and 0% for MET IHC 3+.

**Table 3:** Best overall response (investigator-assessed).

	Phase 1b			Phase 2
	300 mg/day	500 mg/day	Total	500 mg/day
	(*n* = 4)	(*n* = 13)	(*N* = 17)	(*N* = 49)
Best overall response; investigator RECIST v1.1				
CR, *n* (%)	0	0	0	1^a^ (2.0)
PR, *n* (%)	2 (50.0)	0	2 (11.8)	3 (6.1)
SD, *n* (%)	0	4 (30.8)	4 (23.5)	24 (49.0)
PD, *n* (%)	1 (25.0)	8 (61.5)	9 (52.9)	15 (30.6)
Not evaluable, *n* (%)	1 (25.0)	1 (7.7)	2 (11.8)	6 (12.2)
ORR, *n* (%) [90% CI]	2 (50.0) [9.8, 90.2]	0 (0.0) [0.0, 26.0]	2 (11.8) [2.1, 32.6]	4 (8.2) [2.8, 17.7]
DCR, *n* (%) [90% CI]	2 (50.0) [9.8, 90.2]	4 (30.8) [11.3, 57.3]	6 (35.3) [16.6, 58.0]	28 (57.1) [44.4, 69.2]
Best overall response; investigator mRECIST v1.1				
CR, *n* (%)				1^a^ (2.0)
PR, *n* (%)				4 (8.2)
SD, *n* (%)				19 (38.8)
Non-CR/Non-PD, *n* (%)				7 (14.3)
PD, *n* (%)				13 (26.5)
Not evaluable, *n* (%)				5 (10.2)
ORR, *n* (%) [90% CI]				5 (10.2) [4.1, 20.3]
DCR, *n* (%) [90% CI]				24 (49.0) [36.5, 61.5]

*CI* confidence interval, *CR* complete response, *DCR* disease control rate, m*RECIST* modified Response Evaluation Criteria in Solid Tumors, *ORR* objective response rate, *PD* progressive disease, *PR* partial response, *RECIST* Response Evaluation Criteria in Solid Tumors, *SD* stable disease.

^a^CR in a patient with *MET* amplification.

At the time of the analysis, 12 of 17 patients (70.6%) had experienced disease progression and 14 of 17 patients (82.4%) had died. Median TTP was 2.1 months (90% CI: 1.4–7.2), median PFS was 1.5 months (90% CI: 1.4–3.7) and median OS was 7.2 months (90% CI: 3.7–10.1).

**Fig. 1:**
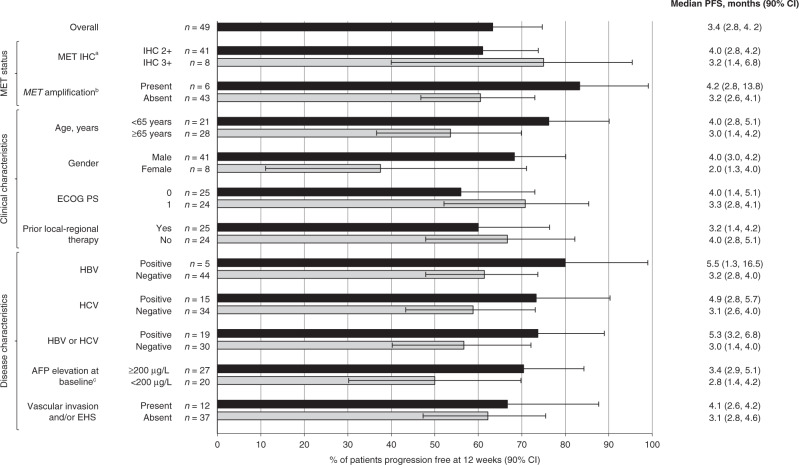
Percentage of patients progression free at 12 weeks according to investigator assessment in Phase 2. Data are shown for the overall population and patient subgroups.^a^Moderate (2+) or strong (3+) staining intensity for MET on IHC in the majority (≥50%) of tumour cells; ^b^*MET* amplification defined as *MET:CEP7* ratio ≥2 or mean gene copy number ≥5; ^c^*AFP* was missing for two patients (12-week PFS in these patients was 100%). *AFP* alpha-fetoprotein; *CI* confidence interval, *EHS* extrahepatic spread, *ECOG PS* Eastern Cooperative Oncology Group performance status, *HBV* hepatitis B virus, *HCV* hepatitis C virus, *IHC* immunohistochemistry, *PFS* progression-free survival.

**Fig. 2:**
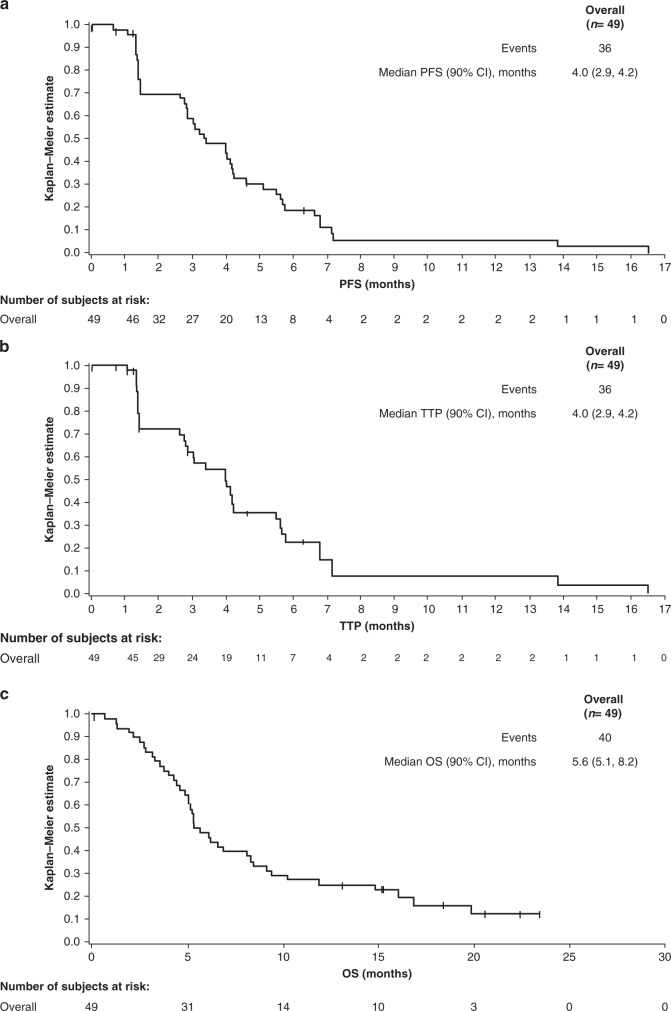
Kaplan–Meier curves for PFS, TTP and OS in Phase 2. **a** Investigator-assessed PFS, **b** investigator-assessed TTP, **c** OS. *CI* confidence interval, *OS* overall survival, *PFS* progression-free survival, *TTP* time to progression.

The primary endpoint of the Phase 2 part of the study was met: 31 of 49 patients (63.3%; 90% CI: 50.5–74.7) were progression-free (as assessed by the investigator) at 12 weeks, which was significantly greater than the 12-week progression-free rate prespecified in the null hypothesis of ≤15% (*P* < 0.0001). PFS at Week 12 was consistent across all subgroups, although there was a trend for better PFS at 12 weeks in patients with MET IHC 3+ (versus 2+), *MET* amplification (versus no *MET* amplification), AFP elevation at baseline ≥200 µg/L (versus <200) and hepatitis B virus/hepatitis C virus (HBV/HCV) positivity (versus HBV/HCV-negative; Fig. 1). Median investigator-assessed PFS was 3.4 months (90% CI: 2.8–4.2; Fig. 2a), and 3.2 months (90% CI: 2–4.6) by independent review committee. Kaplan–Meier curves suggested a trend of improved PFS in patients with HBV/HCV-positive status and possibly in those with *MET* amplification, whereas the PFS curves appeared to be similar irrespective of MET IHC status (Supplementary Fig. 3a, c, e).

**Fig. 3:**
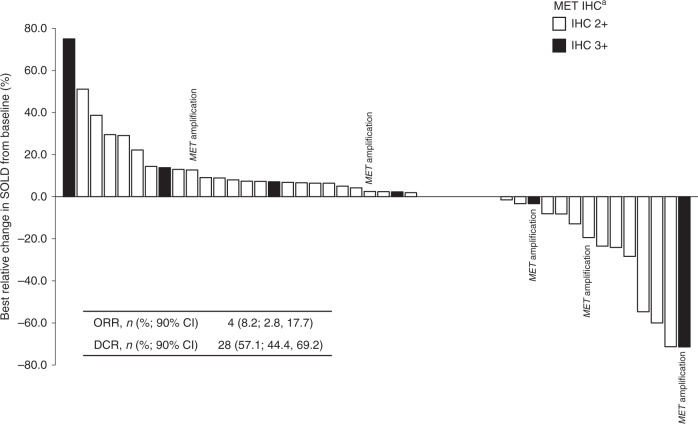
Best relative change in target lesions in Phase 2. Shading indicates MET IHC status: 2+ (white) or 3+ (black). Patients with *MET* amplification are labelled. Inset table shows overall response and disease control rates. ^a^Moderate (2+) or strong (3+) staining intensity for MET on IHC in the majority (≥50%) of tumour cells; *MET* amplification defined as *MET:CEP7* ratio ≥2 or mean gene copy number ≥5. *CI* confidence interval, *DCR* disease control rate, *IHC* immunohistochemistry, *ORR* objective response rate, *SOLD* sum of longest diameter.

Of 49 patients assessed for response, one patient (2.0%) achieved complete response (CR), and three patients (6.1%) achieved PR, giving an investigator-assessed ORR of 4/49 (8.2%; 90% CI: 2.8–17.7). Additionally, 24 patients (49%) had stable disease (SD), resulting in a DCR of 28/49 (57.1%; 90% CI: 44.4–69.2; Fig. 3; Table 3). Of six patients who tested positive for *MET* amplification, one patient (16.7%) achieved CR, four achieved SD (66.7%) and one was not evaluable (16.7%; Fig. 3). Among the eight patients with MET IHC 3+ status, one patient (12.5%) had a CR and four had SD (50%), for a DCR of 62.5%.

Median investigator-assessed TTP was 4.0 months (90% CI: 2.9–4.2; Fig. 2b). As assessed by independent review committee, median TTP was 4.2 months (90% CI: 2.6–5.5). At the time of analysis, 40 patients (81.6%) had died; median OS was 5.6 months (90% CI: 5.1–8.2; Fig. 2c). There was a trend toward improved OS in patients who were HBV- or HCV-positive (versus negative), whereas no clear OS difference was observed with respect to *MET* amplification or MET IHC status (Supplementary Fig. 3b, d, f). Of the 45 patients with baseline and post-baseline AFP assessments, 14 (31.1%) were considered as having a biological response (a decrease in AFP concentration of >20%).

### Pharmacokinetics

In the Phase 1b part of the study, AUC_τ,ss_ was 15,200 ng*h/mL (18.2%) and C_max_ was 734 ng/mL (19.6%) for the 300 mg dose group (values represent the geometric mean and geometric coefficient of variation). Corresponding values for the 500 mg dose group were 12,900 ng*h/mL (50.4%) for AUC_τ,ss_ and 677 ng/mL (44.6%) for C_max_.

## Discussion

The Phase 1b part of the study confirmed the RP2D of tepotinib as 500 mg QD for the treatment of patients with aHCC who had previously received sorafenib. This dosing level is consistent with studies of tepotinib in other tumour types, and in Asian patients with HCC with MET overexpression.^24,28,30^ Preliminary anti-tumour activity was observed in Phase 1b with tepotinib: two patients achieved PR and four patients achieved SD. Following results from Phase 1b, Phase 2 investigated the efficacy and safety of tepotinib dosed at 500 mg QD in patients with HCC tumours with MET overexpression who had previously been treated with sorafenib. The Phase 2 primary endpoint was met: 63.3% of patients remained alive and progression-free according to investigator- assessment at 12 weeks, which was significantly greater than the ≤15% rate prespecified in null hypothesis (based on historical data).^23^ Median PFS was 3.4 months, median TTP was 4.0 months (both investigator-assessed) and median OS was 5.6 months.

Tepotinib was generally well tolerated, with no new safety signals identified. No DLTs were reported in Phase 1b, and treatment-related AEs were reported in 82.4% of patients overall, and at Grade ≥3 in 23.5%. The safety profile observed in Phase 2 was consistent with Phase 1b and with other studies of tepotinib.^24,25,28,30^ Treatment-related AEs were reported in 83.7% of patients overall and at Grade ≥3 in 28.6%. The most common Grade ≥3 treatment-related AEs were peripheral oedema and increased lipase, which are both known safety signals with tepotinib.^24^ The safety profile is encouraging, especially given that some patients received treatment for >15 months, and makes tepotinib particularly suited for investigation in combination with other classes of agent such as immunotherapies.

Although no clear differences in efficacy of tepotinib according to MET status were evident, 12-week PFS rates were numerically higher in patients with MET IHC 3+ staining (versus 2+) or *MET* amplification (versus no amplification). The DCR also appeared more favourable among patients with *MET* amplification relative to the overall population. Prior reports have suggested that MET overexpression/amplification status may predict response to MET inhibition.^18,31,32^ For example, in a Phase 2 study of the MET inhibitor capmatinib in aHCC, objective response was observed in 3/30 patients (10%) with a less stringent definition of *MET* alteration (MET H-score ≥50%, *MET*:*CEP7* ratio ≥2 or *MET* gene copy number ≥5; *n* = 30), and 3/10 patients (30%) when a more strict definition of *MET* alteration was applied (MET IHC 3+, or 2+ with *MET* gene copy number ≥5; *n* = 10).^31^

We also observed a trend for improved PFS and OS in patients who were HBV/HCV-positive versus negative. This may reflect a positive prognostic effect, as both HBV- and HCV-associated HCC have been independently associated with longer survival compared with HCC of other aetiologies in recent analyses.^33,34^ Interestingly, we also noted a trend for greater 12-week PFS in patients with AFP elevation at baseline ≥200 µg/L (versus <200 µg/L). In addition to its role as a poor prognostic factor,^35^ increased AFP levels are an established positive predictive marker for ramucirumab, which has been approved for second-line use specifically in patients with AFP elevation following results from the Phase 3 REACH-2 trial.^11^ Higher AFP levels were also associated with a more pronounced benefit of the multikinase inhibitor cabozantinib versus placebo in the randomised Phase 3 CELESTIAL trial.^36^

Exposure in this HCC population, as derived from the rich PK sampling in the Phase 1b part, was lower than in the first-in-human trial in patients with advanced solid tumours (47% of the AUC_τ,ss_ and 52% of the C_max_).^24^ A similar reduction in exposure was observed in the first-line Phase 1b/2 tepotinib trial in HCC.^37^ This is in line with findings from a population PK analysis showing that liver cirrhosis is associated with lower exposure to tepotinib and a dedicated PK hepatic impairment trial demonstrating a reduction in exposure (of 12% for AUC from time 0 to infinity and 29% for C_max_) in patients with moderate (Child–Pugh B) hepatic impairment relative to control patients.^38^

In sorafenib pretreated aHCC, MET overexpression is a poor prognostic factor that has been associated with significantly shorter median OS (3.8 months) compared with tumours without MET overexpression (9.0 months).^23^ Despite enrolment of this poor prognostic group, median PFS in the present study appears to be similar to that reported in second-line Phase 3 trials in MET-unselected tumours of regorafenib,^10^ ramucirumab^39^ and pembrolizumab,^15^ although it is shorter than reported with cabozantinib.^12^ The negative prognostic effect of MET overexpression is likely to explain the lower median OS with tepotinib relative to even the placebo arms of those trials. Although longer OS has been reported in sorafenib pretreated aHCC with MET overexpression in two tivantinib Phase 3 trials,^40,41^ those results may not be directly comparable with our trial for two reasons. First, MET was assessed in archival specimens in a substantial proportion of patients in the tivantinib trials and so might not have indicated MET status at the time of enrolment. Second, turnaround time for central IHC assessment in the tivantinib trials (median of 43 days for METIV-HCC)^40^ could have led to exclusion of patients with more aggressive disease due to rapid progression or declining performance status while awaiting central MET status determination. In contrast, eligibility for the tepotinib Phase 2 trial required demonstration of MET overexpression in a biopsy acquired within 28 days of the first study dose, thereby minimising the potential for evolution in MET status or loss of patients with aggressive disease during assessment for eligibility.

In addition to these new second-line options, the first-line HCC treatment landscape has evolved with the approval of the multikinase inhibitor lenvatinib and the immunotherapy atezolizumab (in combination with bevacizumab) for the initial treatment of unresectable HCC following positive results from the Phase 3 trials.^9,13,42^ Although immunotherapy trials in HCC have not been universally positive,^15,43^ immunotherapy-based combinations are a promising avenue of investigation, with several ongoing trials investigating regimens consisting of an immune checkpoint blocker with a tyrosine kinase inhibitor.^44^ Of note, the immunosuppressive function of the HGF/MET pathway provides a preclinical rationale for combining MET inhibitors with programmed death-1/programmed death-ligand 1 inhibitors.^45^ A Phase 3 trial of first-line cabozantinib plus atezolizumab in aHCC is currently underway.^46^

How novel first-line therapies may impact the activity of second-line tepotinib demonstrated in the present study is unknown. Other limitations include the single-arm, non-randomised design and the relatively small number of enrolled patients with MET IHC 3+ staining or *MET* amplification, which preclude definitive conclusions regarding the potential predictive relevance of MET-based biomarkers in this study, especially in Phase 1b. Finally, efficacy outcomes cannot be compared directly between the two phases of the study due to differences in MET status (unselected in Phase 1b vs IHC 2+/3+ in Phase 2), baseline characteristics (higher proportion of patients aged <65 years in Phase 2), tepotinib dose and sample size.

In both phases of the study, tepotinib was generally well tolerated by Western patients with aHCC who had previously received sorafenib treatment. At the RP2D (500 mg QD), tepotinib showed promising efficacy and, therefore, a positive benefit–risk balance for patients with aHCC with MET overexpression who had previously received sorafenib therapy.

## Supplementary information

Supplementary information

## Data Availability

Data are held by the sponsor, Merck KGaA (Darmstadt, Germany), to whom any request for additional data should be addressed.

## References

[1] Bray F, Ferlay J, Soerjomataram I, Siegel RL, Torre LA, Jemal A (2018). Global cancer statistics 2018: GLOBOCAN estimates of incidence and mortality worldwide for 36 cancers in 185 countries. CA Cancer J. Clin..

[2] Llovet JM, Zucman-Rossi J, Pikarsky E, Sangro B, Schwartz M, Sherman M (2016). Hepatocellular carcinoma. Nat. Rev. Dis. Prim..

[3] Yang JD, Hainaut P, Gores GJ, Amadou A, Plymoth A, Roberts LR (2019). A global view of hepatocellular carcinoma: trends, risk, prevention and management. Nat. Rev. Gastroenterol. Hepatol..

[4] Park JW, Chen M, Colombo M, Roberts LR, Schwartz M, Chen PJ (2015). Global patterns of hepatocellular carcinoma management from diagnosis to death: the BRIDGE Study. Liver Int..

[5] European Association for the Study of the Liver. EASL Clinical Practice Guidelines: Management of hepatocellular carcinoma. *J. Hepatol*. **69**, 182–236 (2018). 10.1016/j.jhep.2018.03.019 29628281

[6] Bruix J, da Fonseca LG, Reig M (2019). Insights into the success and failure of systemic therapy for hepatocellular carcinoma.. Nat. Rev. Gastroenterol. Hepatol..

[7] Llovet JM, Ricci S, Mazzaferro V, Hilgard P, Gane E, Blanc JF (2008). Sorafenib in advanced hepatocellular carcinoma. N. Engl. J. Med..

[8] Cheng A-L, Kang Y-K, Chen Z, Tsao C-J, Qin S, Kim JS (2009). Efficacy and safety of sorafenib in patients in the Asia-Pacific region with advanced hepatocellular carcinoma: a phase 3 randomised, double-blind, placebo-controlled trial. Lancet Oncol..

[9] Kudo M, Finn RS, Qin S, Han KH, Ikeda K, Piscaglia F (2018). Lenvatinib versus sorafenib in first-line treatment of patients with unresectable hepatocellular carcinoma: a randomised phase 3 non-inferiority trial. Lancet.

[10] Bruix J, Qin S, Merle P, Granito A, Huang YH, Bodoky G (2017). Regorafenib for patients with hepatocellular carcinoma who progressed on sorafenib treatment (RESORCE): a randomised, double-blind, placebo-controlled, phase 3 trial. Lancet.

[11] Kelley RK, Cheng A-L, Braiteh FS, Park J-W, Benzaghou F, Milwee S (2019). Phase 3 (COSMIC-312) study of cabozantinib (C) in combination with atezolizumab (A) versus sorafenib (S) in patients (pts) with advanced hepatocellular carcinoma (aHCC) who have not received previous systemic anticancer therapy. J. Clin. Oncol..

[12] Abou-Alfa GK, Meyer T, Cheng A-L, El-Khoueiry AB, Rimassa L, Ryoo B-Y (2018). Cabozantinib in patients with advanced and progressing hepatocellular carcinoma. N. Engl. J. Med..

[13] Finn RS, Qin S, Ikeda M, Galle PR, Ducreux M, Kim TY (2020). Atezolizumab plus bevacizumab in unresectable hepatocellular carcinoma. N. Engl. J. Med..

[14] Zhu AX, Finn RS, Edeline J, Cattan S, Ogasawara S, Palmer D (2018). Pembrolizumab in patients with advanced hepatocellular carcinoma previously treated with sorafenib (KEYNOTE-224): a non-randomised, open-label phase 2 trial. Lancet Oncol..

[15] Finn RS, Ryoo B-Y, Merle P, Kudo M, Bouattour M, Lim HY (2020). Pembrolizumab as second-line therapy in patients with advanced hepatocellular carcinoma in KEYNOTE-240: a randomized, double-blind, phase 3 trial. J. Clin. Oncol..

[16] El-Khoueiry AB, Sangro B, Yau T, Crocenzi TS, Kudo M, Hsu C (2017). Nivolumab in patients with advanced hepatocellular carcinoma (CheckMate 040): an open-label, non-comparative, phase 1/2 dose escalation and expansion trial. Lancet.

[17] Yau T, Kang Y-K, Kim T-Y, El-Khoueiry AB, Santoro A, Sangro B (2020). Efficacy and safety of nivolumab plus ipilimumab in patients with advanced hepatocellular carcinoma previously treated with sorafenib: the CheckMate 040 randomized clinical trial. JAMA Oncol..

[18] Bouattour M, Raymond E, Qin S, Cheng A-L, Stammberger U, Locatelli G (2018). Recent developments of c-Met as a therapeutic target in hepatocellular carcinoma. Hepatology.

[19] Rimassa L, Abbadessa G, Personeni N, Porta C, Borbath I, Daniele B (2016). Tumor and circulating biomarkers in patients with second-line hepatocellular carcinoma from the randomized phase 2 study with tivantinib. Oncotarget.

[20] Cascone T, Xu L, Lin HY, Liu W, Tran HT, Liu Y (2017). The HGF/c-MET pathway is a driver and biomarker of VEGFR-inhibitor resistance and vascular remodeling in non-small cell lung cancer. Clin. Cancer Res..

[21] Daudigeos-Dubus E, Le Dret L, Bawa O, Opolon P, Vievard A, Villa I (2017). Dual inhibition using cabozantinib overcomes HGF/MET signaling mediated resistance to pan-VEGFR inhibition in orthotopic and metastatic neuroblastoma tumors. Int. J. Oncol..

[22] Zhou L, Liu XD, Sun M, Zhang X, German P, Bai S (2016). Targeting MET and AXL overcomes resistance to sunitinib therapy in renal cell carcinoma. Oncogene.

[23] Santoro A, Rimassa L, Borbath I, Daniele B, Salvagni S, Van Laethem JL (2013). Tivantinib for second-line treatment of advanced hepatocellular carcinoma: a randomised, placebo-controlled phase 2 study. Lancet Oncol..

[24] Falchook GS, Kurzrock R, Amin HM, Xiong W, Fu S, Piha-Paul SA (2020). First-in-man phase 1 trial of the selective MET inhibitor tepotinib in patients with advanced solid tumors. Clin. Cancer Res..

[25] Paik PK, Felip E, Veillon R, Sakai H, Cortot AB, Garassino MC (2020). Tepotinib in non-small-cell lung cancer with MET exon 14 skipping mutations. N. Engl. J. Med..

[26] Bladt F, Friese-Hamim M, Ihling C, Wilm C, Blaukat A (2014). The c-Met inhibitor MSC2156119J effectively inhibits tumor growth in liver cancer models.. Cancers (Basel).

[27] Wu YL, Cheng Y, Zhou J, Lu S, Zhang Y, Zhao J (2020). Tepotinib plus gefitinib in patients with EGFR-mutant non-small-cell lung cancer with MET overexpression or MET amplification and acquired resistance to previous EGFR inhibitor (INSIGHT study): an open-label, phase 1b/2, multicentre, randomised trial. Lancet Res. Med..

[28] Ryoo BY, Ren Z, Kim TY, Pan H, Rau KM, Choi HJ (2018). Phase 2 trial of tepotinib vs sorafenib in Asian patients (pts) with advanced hepatocellular carcinoma (HCC).. Ann. Oncol..

[29] Lencioni R, Llovet JM (2010). Modified RECIST (mRECIST) assessment for hepatocellular carcinoma. Semin. Liver Dis..

[30] Shitara K, Yamazaki K, Tsushima T, Naito T, Matsubara N, Watanabe M (2020). Phase 1 trial of the MET inhibitor tepotinib in Japanese patients with solid tumors. Jpn. J. Clin. Oncol.

[31] Qin S, Chan SL, Sukeepaisarnjaroen W, Han G, Choo SP, Sriuranpong V (2019). A phase 2 study of the efficacy and safety of the MET inhibitor capmatinib (INC280) in patients with advanced hepatocellular carcinoma. Ther. Adv. Med. Oncol..

[32] Moosavi F, Giovannetti E, Saso L, Firuzi O (2019). HGF/MET pathway aberrations as diagnostic, prognostic, and predictive biomarkers in human cancers. Crit. Rev. Clin. Lab. Sci..

[33] Noda Y, Kawaguchi T, Kuromatsu R, Komukai S, Nakano M, Niizeki T (2019). Prognostic profile of patients with non-viral hepatocellular carcinoma: A comparative study with hepatitis C virus-related hepatocellular carcinoma using data mining analysis. Oncol. Lett..

[34] Brar G, McNeel T, McGlynn K, Graubard B, Floudas C, Pia Morelli M (2019). Hepatocellular carcinoma (HCC) survival by etiology: A SEER-Medicare database analysis. J. Clin. Oncol..

[35] Bai D-S, Zhang C, Chen P, Jin S-J, Jiang G-Q (2017). The prognostic correlation of AFP level at diagnosis with pathological grade, progression, and survival of patients with hepatocellular carcinoma. Sci. Rep..

[36] Kelley RK, Meyer T, Rimassa L, Merle P, Park J-W, Yau T (2020). Serum alpha-fetoprotein levels and clinical outcomes in the Phase 3 CELESTIAL study of cabozantinib versus placebo in patients with advanced hepatocellular carcinoma.. Clin. Cancer Res..

[37] Ryoo, B.-Y., Cheng, A.-L., Ren, Z., Kim, T.-Y. Pan, H., Rau, K.-M. et al. Randomised Phase 1b/2 trial of tepotinib vs sorafenib in Asian patients with advanced hepatocellular carcinoma with MET overexpression. *Br J Cancer*. 10.1038/s41416-021-01380-3 (2021). 10.1038/s41416-021-01380-3 PMC8292411 33972742

[38] TEPMETKO® (tepotinib) Japanese Package Insert. March 2020 revision.

[39] Zhu AX, Park JO, Ryoo BY, Yen CJ, Poon R, Pastorelli D (2015). Ramucirumab versus placebo as second-line treatment in patients with advanced hepatocellular carcinoma following first-line therapy with sorafenib (REACH): a randomised, double-blind, multicentre, phase 3 trial. Lancet Oncol..

[40] Rimassa L, Assenat E, Peck-Radosavljevic M, Pracht M, Zagonel V, Mathurin P (2018). Tivantinib for second-line treatment of MET-high, advanced hepatocellular carcinoma (METIV-HCC): a final analysis of a phase 3, randomised, placebo-controlled study. Lancet Oncol..

[41] Kudo M, Morimoto M, Moriguchi M, Izumi N, Takayama T, Yoshiji H (2020). A randomized, double-blind, placebo-controlled, phase 3 study of tivantinib in Japanese patients with MET-high hepatocellular carcinoma. Cancer Sci..

[42] TECENTRIQ® (atezolizumab) US Prescribing Information. November 2020 revision.

[43] Yau T, Park J, Finn R, Cheng AL, Mathurin P, Edeline J (2019). CheckMate 459: a randomized, multi-center phase 3 study of nivolumab (NIVO) vs sorafenib (SOR) as first-line (1L) treatment in patients (pts) with advanced hepatocellular carcinoma (aHCC). Ann. Oncol..

[44] Cheng H, Sun G, Chen H, Li Y, Han Z, Zhang P (2019). Trends in the treatment of advanced hepatocellular carcinoma: immune checkpoint blockade immunotherapy and related combination therapies. Am. J. Cancer Res..

[45] Papaccio F, Della Corte CM, Viscardi G, Di Liello R, Esposito G, Sparano F (2018). HGF/MET and the immune system: relevance for cancer immunotherapy. Int. J. Mol. Sci..

[46] Kelley RK, J WO, Hazra S, Benzaghou F, Yau T, Cheng AL (2020). Cabozantinib in combination with atezolizumab versus sorafenib in treatment-naive advanced hepatocellular carcinoma: COSMIC-312 Phase 3 study design. Future Oncol..

