# Correction: Phase 1b/2 trial of tepotinib in sorafenib pretreated advanced hepatocellular carcinoma with MET overexpression

**DOI:** 10.1038/s41416-021-01403-z

**Published:** 2021-06-02

**Authors:** Thomas Decaens, Carlo Barone, Eric Assenat, Martin Wermke, Angelica Fasolo, Philippe Merle, Jean-Frédéric Blanc, Véronique Grando, Angelo Iacobellis, Erica Villa, Joerg Trojan, Josef Straub, Rolf Bruns, Karin Berghoff, Juergen Scheele, Eric Raymond, Sandrine Faivre

**Affiliations:** 1 University Grenoble Alpes, Department of Hepato-Gastroenterology and Digestive Oncology, CHU Grenoble Alpes, Institute for Advanced Biosciences INSERM U1209, Grenoble, France; 2 Medical Oncology, Policlinico Universitario A. Gemelli, Roma, Italy; 3 Medical Oncology, CHU Saint Eloi, Montpellier, France; 4 NCT/UCC Early Clinical Trial Unit, University Hospital Carl-Gustav-Carus, Dresden, Germany; 5 Oncology, IRCCS San Raffaele, Milan, Italy; 6 Service d’Hépato-Gastro-Entérologie, Hôpital de la Croix Rousse, Lyon, France; 7 Service d’Hépato-Gastroentérologie et d’Oncologie Digestive, Groupe Hospitalier Haut-Lévêque, CHU Bordeaux, Pessac, France; 8 Service Hépatologie, Hôpital Jean-Verdier, Bondy, France; 9 Reparto di Gastroenterologia ed Endoscopia Digestiva, Ospedale Casa Sollievo della Sofferenza IRCCS, San Giovanni Rotondo, Italy; 10 Division of Gastroenterology Policlinico di Modena, Modena, Italy; 11 Gastrointestinal Oncology, Goethe University Hospital, Frankfurt, Germany; 12 Clinical Biomarker & Companion Diagnostics, Merck KGaA, Darmstadt, Germany; 13 Biostatistics, Merck KGaA, Darmstadt, Germany; 14 Global Patient Safety Innovation, Merck KGaA, Darmstadt, Germany; 15 Global Clinical Development Oncology, Merck KGaA, Darmstadt, Germany; 16 Medical Oncology, Paris-St Joseph Hospital, Paris, France; 17 Medical Oncology, Saint-Louis Hospital & Paris 7 University, Paris, France

**Keywords:** Hepatocellular carcinoma, Molecularly targeted therapy

Correction to: *British Journal of Cancer* 10.1038/s41416-021-01334-9, published online 6 April 2021

The original version of this article unfortunately contained a mistake. Due to a typesetting error ‘sorafenib pretreated’ was changed to ‘sorafenibpretreated’ throughout the article. The correct spelling is ‘sorafenib pretreated. There were also minor errors in Fig. 1 and Fig. 3. The original article has been corrected.

