# Supplementary material for: Phase 1b/2 trial of tepotinib in sorafenib pretreated advanced hepatocellular carcinoma with MET overexpression
Source: Br J Cancer. 2021 Apr 6;125(2):190–9. doi: 10.1038/s41416-021-01334-9 (PMC8292404; doi:10.1038/s41416-021-01334-9)
Supplement: Supplementary file 1 — Supplementary information [file 41416_2021_1334_MOESM1_ESM.docx]

# SUPPLEMENTARY INFORMATION

**Supplementary Table 1.** Study inclusion and exclusion criteria

| **Inclusion criteria** | **Exclusion criteria** |
| --- | --- |
| For inclusion in the study, all of the following criteria had to be fulfilled:   1. Histologically confirmed HCC 2. Child–Pugh Class A liver function score 3. MET overexpression status, as determined by the central laboratory (in Phase II prospectively for patient selection), defined as MET protein overexpression (moderate [2+] or strong [3+] staining intensity for MET on IHC in the majority [≥50%] of tumour cells) 4. Availability of a pretreatment tumour biopsy (excluding fine needle aspiration and cytology samples) taken after the patient had discontinued sorafenib and within 28 days before the day of first dosing with tepotinib 5. Male or female, 18 years of age or older 6. Measurable disease in accordance with RECIST Version 1.1 7. ECOG PS 0 to 1 (inclusive) 8. Previously treated with sorafenib for ≥4 weeks and discontinued sorafenib treatment at least 14 days prior to day 1 due to either intolerance or radiographic progression 9. Signed and dated informed consent indicating that the patient had been informed of all the pertinent aspects of the study prior to enrollment 10. Willingness and ability to comply with scheduled visits, treatment plans, laboratory tests and other study procedures 11. Life expectancy of at least 3 months as judged by the investigator | Patients were not eligible if they fulfilled any of the following criteria:   1. Prior systemic anticancer treatment for aHCC (except for sorafenib as described in the inclusion criteria) 2. Prior treatment with any agent targeting the HGF/MET pathway 3. Local regional therapy within 4 weeks before day 1 (e.g. surgery, radiation therapy, hepatic arterial embolisation, transcatheter arterial chemoembolisation, chemoembolisation, radiofrequency ablation, percutaneous ethanol injection and cryoablation) 4. Laboratory index at baseline: haemoglobin ≤8.5 g/dL; neutrophils  <1.5 × 10^9^/L; platelets <60 × 10^9^/L; TBL >3 mg/dL; AST or ALT >5 × ULN; serum creatinine ≥1.5 × ULN; calculated CrCl <60 mL/min according to the Cockcroft–Gault formula; international normalised ratio >2.3 5. Past or current history of neoplasm other than HCC, except for curatively treated non-melanoma skin cancer, *in situ* carcinoma of the cervix, or other cancer curatively treated and with no evidence of disease for at least 5 years 6. Known central nervous system or brain metastasis that was either symptomatic or untreated 7. Medical history of difficulty in swallowing, malabsorption or other chronic gastrointestinal disease, or conditions that may have hampered compliance and/or absorption of the tested products 8. Clinically significant gastrointestinal bleeding within 4 weeks before study entry 9. Peripheral neuropathy grade ≥2 (CTCAE Version 4.0) 10. Impaired cardiac function evidenced by any of the following conditions: left ventricular ejection fraction <45% defined by echocardiograph (screening assessment was not required for patients without a history of congestive heart failure unless clinically indicated); serious arrhythmia; unstable angina pectoris; myocardial infarction within the last 12 months prior to study entry, or pericardial effusion; QTc prolongation >470 ms, risk factors for Torsades de Pointes (Heart Insufficiency NYHA II–IV); hypokalaemia, family history of long QT syndrome 11. Uncontrolled hypertension by standard medication (not stabilised to 150/90 mmHg or below) 12. Known HIV infection 13. Known or suspected drug hypersensitivity to any ingredients of tepotinib 14. Pregnancy, lactation or patients not of childbearing potential due to being postmenopausal (female patients only), or patients of reproductive potential not willing or able to employ a highly effective method of birth control/contraception to prevent pregnancy from screening until 3 months after receiving the last dose of study drug. A highly effective method of contraception was defined as having a low failure rate (<1% per year) when used consistently and correctly 15. Concurrent treatment with non-permitted drug 16. Substance abuse, other acute or chronic medical or psychiatric condition or laboratory abnormalities that might have increased the risk associated with study participation at the discretion of investigators 17. Prior treatment with tepotinib or other c-Met inhibitors 18. Participation in another interventional clinical study within the 28 days prior to day 1 19. Previous anticancer treatment-related toxicities not recovered to grade 0 to 1 or baseline (except alopecia, peripheral neuropathy, and elevated liver enzymes) 20. History of liver transplant 21. Active or uncontrolled infections except chronic HBV, chronic HCV, or both 22. Any concurrent medical condition or disease that would potentially compromise the conduct of the study (at the discretion of the investigators) |

*aHCC* advanced hepatocellular carcinoma, *ALT* alanine transaminase, *AST* aspartate transaminase, *CrCl* creatinine clearance, *CTCAE* Common Terminology Criteria for Adverse Events, *ECOG PS* Eastern Cooperative Oncology Group performance status, *HBV* hepatitis B virus, *HCV* hepatitis C virus, *HCC* hepatocellular carcinoma, *HGF* hepatocyte growth factor, *HIV* human immunodeficiency virus, *IHC* immunohistochemistry, *NYHA* New York Heart Association, *RECIST* Response Evaluation Criteria in Solid Tumors, *TBL* total bilirubin, *ULN* upper limit of normal.

**Supplementary Table 2.** MET IHC antibody

| **Antibody** | **Clone** | **Manufacturer** | **Quality control** |
| --- | --- | --- | --- |
| Dako pharmDx anti-total MET rabbit monoclonal antibody | D1C2 | Dako, Agilent Technologies, Inc., Santa Clara, CA | Each MET IHC assay had positive and negative tissue controls, as well as a negative reagent control for each specimen that was included in each staining run. If there was failure of any one of the controls, patient results were considered to be invalid and the test re-run. The pathologist reviewed all the control slides prior to scoring patient samples. |

*IHC* immunohistochemistry.

## **Supplementary Table 3.** DLT criteria in Phase Ib

| DLT criteria |
| --- |
| Any of the following toxicities at any dose level and judged to be related to trial treatment by the investigator:   - Grade 4 neutropenia for >7 days - Grade ≥3 febrile neutropenia for >1 day - Grade 4 thrombocytopenia or grade 3 with non-traumatic bleeding - Grade ≥3 nausea/vomiting and/or diarrhoea despite optimal treatment for more than 3 days - Any grade ≥3 non-haematological adverse event except aforementioned gastrointestinal events and alopecia. Specific definitions existed for the following cases:   - Grade ≥3 liver adverse event requiring a recovery period of more than 7 days to the baseline or to grade 1 (or less; this criterion was not limited to liver function tests)   - Grade ≥3 lipase and/or amylase elevation with confirmation of pancreatitis, either based on clinical or radiological signs, was not considered to be a DLT. An isolated lipase and/or amylase elevation of grade ≥3 without clinical or radiological evidence of pancreatitis was not classified as a DLT |

*DLT* dose-limiting toxicity.

## **Supplementary Table 4.** List of institutional review boards or independent ethics committees of each centre

| **Site #** | **Investigator name** | **IRB/IEC name** | **Chairperson (if known)** | **IRB reference number** |
| --- | --- | --- | --- | --- |
| 0102 | Dr Sven Francque | Central Ethics Committee  Ethisch Comité Universitair  Ziekenhuis Antwerpen  Wilrijkstraat 10  2650 Edegem  Belgium | Prof. Dr Patric Cras | Not applicable |
| 0103 | Dr Hans Van Vlierberghe | Local Ethics Committee  Ethisch Comité Universitair  Ziekenhuis Gent De Pintelaan 1851K4  9000 Gent  Belgium | Not Available |  |
|  |  | Central Ethics Committee  Ethisch Comité Universitair  Ziekenhuis Antwerpen  Wilrijkstraat 10  2650 Edegem  Belgium | Prof. Dr Patric Cras |  |
| 0201 | Dr Sandrine Faivre | Central Ethics Committee  Comité de Protection de Personnes Ile de France VI  47-83 Boulevard de l'Hôpital  France | Prof. Nathalie Brion | Not applicable |
| 0202 | Dr Philippe Merle |  |  |  |
| 0203 | Dr Jean-Frederic Blanc |  |  |  |
| 0204 | Dr Véronique Grando |  |  |  |
| 0205 | Dr Laetitia Fartoux |  |  |  |
| 0206 | Dr Stéphane Cattan |  |  |  |
| 0207 | Dr Jean-Pierre Bronowicki |  |  |  |
| 0208 | Dr Helene Regnault |  |  |  |
| 0209 | Dr Thomas Decaens |  |  |  |
| 0210 | Dr Eric Assenat |  |  |  |
| 0211 | Dr Nicolas Isambert |  |  |  |
| 0303 | Dr Marcus Woerns | Local Ethics Committee  Ethik-Kommission bei der Landesärztekammer Rheinland-Pfalz  Deutschhausplatz 3  55116 Mainz  Germany | Not Available | DE/EKRP36 |
| 0306 | Dr Michael Schultheiss | Local Ethics Committee  Ethik-Kommission der Albert-Ludwigs-Universität Freiburg  Engelberger Str. 21  79106 Freiburg  Germany | Not Available | DE/EKBW02 |
| 0307 | Dr Martin Wermke | Local Ethics Committee  Ethikkommission an der TU Dresden  Fetscherstr. 74  01307 Dresden  Germany | Not Available | DE/EKSN38 |
| 0303 | Dr Marcus Woerns | Central Ethics Committee  Ethik-Kommission des Fachbereichs  Medizin der Johann Wolfgang Goethe-  Theodor-Stern-Kai 7  Studienzentrale der Med. Klinik II  Haus 11, 3. OG  Germany | Not Available | DE/EKHE18 |
| 0304 | Dr Joerg Trojan |  |  |  |
| 0306 | Dr Michael Schultheiss |  |  |  |
| 0307 | Dr Martin Wermke |  |  |  |
| 0401 | Dr Antonio Avallone | Local Ethics Committee  Istituto Nazionale Tumori– IRCCS Fondazione G. Pascale  Via Mariano 53, Semmola 80131 Napoli NA  Italy | Not Available | CE150093 |
| 0402 | Dr Erica Villa | Local Ethics Committee  Comitato Etico Provinciale di Modena  Via de Pozzo 71,  41124 Modena  Italy | Not Available | CE150191 |
| 0403 | Dr Pietro Lampertico | Local Ethics Committee  Comitato Etico Milano Area B Via Francesco Sforza 28  20122 Milano  Italy | Ferruccio Berti | CE150176 |
| 0407 | Dr Carlo Barone | Local Ethics Committee  Comitato Etico dell'Università  Cattolica del Sacro Cuore e annesso  Policlinico "A. Gemelli" Largo  Agostino Gemelli, 8 AOU Senese – Policlinico Agostino Gemelli  00168 Roma  Italy | Not Available | CE150057 |
| 0408 | Dr Luca Gianni | Local Ethics Committee  Comitato Etico dell'IRCCS Ospedale  S. Raffaele di Milano  Via Olgettina, 60  20132 Milano  Italy | Not Available | CE150050 |
| 0409 | Dr Bruno Daniele | Local Ethics Committee  Comitato Etico Campania Nord  Azienda Ospedaliera “San Giuseppe  Moscati” di Avellino -Contrada Amoretta - Citta Ospedaliera  83100 Avellino  Italy | Not Available | CE150128 |
| 0405 | Dr Rossana Berardi | Local Ethics Committee  Comitato Etico Regionale (C.E.R.) delle Marche Ospedali Riuniti  Umberto I-G.M. Lancisi-G. Salesi di Ancona  Via Conca, 71 60126 Torrette di Ancona  Italy | Not Available | CE150164 |
| 0410 | Dr Angelo Iacobellis | Local Ethics Committee  Sezione del Comitato Etico IRCCS  Instituto Tumori Giovanni Paolo II di  Bari presso la Fondazione Casa  Sollievo della Sofferenza Viale Cappuccini  71013 San Giovanni Rotondo  Italy | Not Available | CE150037 |
| 0401 | Dr Antonio Avallone | Local and Central Ethics Committee  Comitato Etico Milano Area B Via Francesco Sforza 28  20122 Milano Italy | Not Available | CE150176 |
| 0402 | Dr Erica Villa |  |  |  |
| 0403 | Dr Pietro Lampertico |  |  |  |
| 0405 | Dr Rossana Berardi |  |  |  |
| 0407 | Dr Carlo Barone |  |  |  |
| 0408 | Dr Luca Gianni |  |  |  |
| 0409 | Dr Bruno Daniele |  |  |  |
| 0410 | Dr Angelo Iacobellis |  |  |  |
| 0501 | Dr Juan Delgado Mingorance | Central Ethics Committee  CEIC Grupo HM  Hospital Universitario Madrid Montepríncipe  Avda. Monteprínicpe Nº 25  Spain | Not Available | Not applicable |
| 0502 | Dr Antonio Cubillo Gracian |  |  |  |
| 0503 | Dr Andres Muñoz Martin |  |  |  |
| 0703 | Dr Martin Gutierrez | Local Ethics Committee  Western Institutional Review Board  1019 39th Avenue SE Suite 120 Puyallup  WA 98374-2115  USA | Not Available | IRB00000533 (registration n°) + parent organiz N° IORG0000432 |
| 0706 | Dr Ari Baron |  |  |  |
| 0707 | Dr Angel Alsina |  |  |  |
| 0708 | Dr Anne Noonan |  |  |  |
| 0701 | Dr Richy Agajanian | Central Ethics Committee  Copernicus Group IRB  5000 CentreGreen Way  Suite 200  Cary, NC 27513  USA | Not Available | IRB00000533 (registration n°) + parent organiz N° IORG0000433 |
| 0702 | Dr Julio Peguero |  |  |  |
| 0703 | Dr Martin Gutierrez |  |  |  |
| 0706 | Dr Ari Baron |  |  |  |
| 0707 | Dr Angel Alsina |  |  |  |
| 0708 | Dr Anne Noonan |  |  |  |

## IEB independent ethics committee, IRB institutional review board.

## **Supplementary Fig. 1.** Phase II: Study design


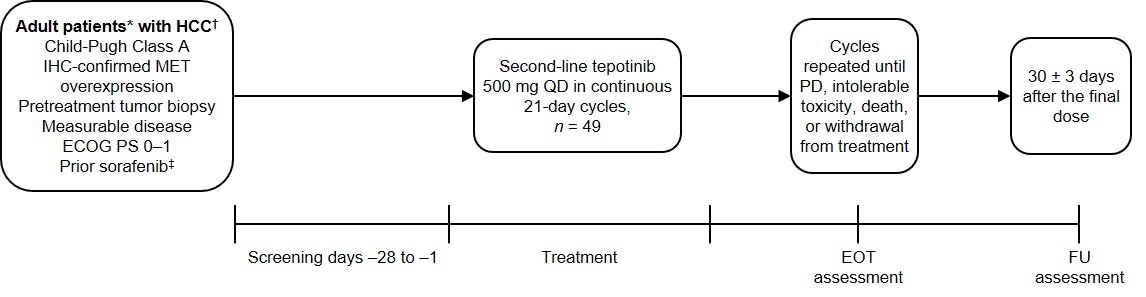


^*^From 22 sites in Belgium, France, Germany, Italy, Spain, and the US; ^†^Histologically confirmed; ^‡^Previously treated with sorafenib for ≥4 weeks and discontinued sorafenib treatment ≥14 days prior to day 1, due to either intolerance or radiographic progression.

*ECOG PS* Eastern Cooperative Oncology Group performance status, *EOT* end of treatment, *FU* follow-up, *HCC* hepatocellular carcinoma, *IHC* immunohistochemistry, *PD* progressive disease, *QD* once daily.

## **Supplementary Fig. 2.** Patient disposition in Phase Ib (**a**) and Phase II (**b**)

**a.**

**
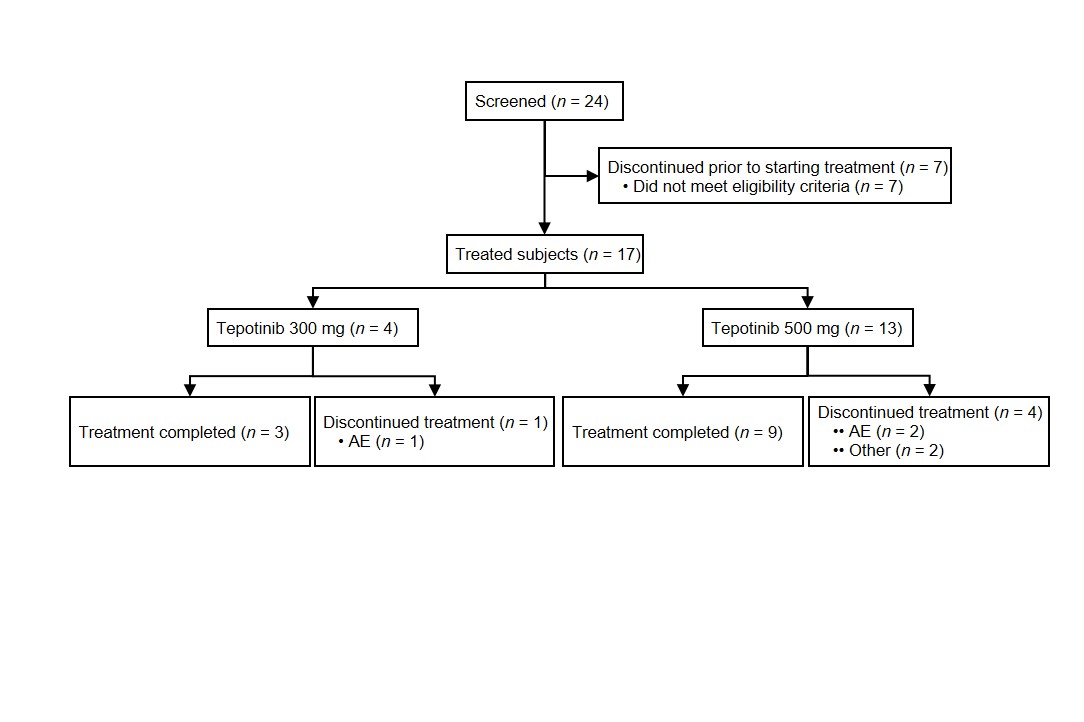
**

**b.**

**
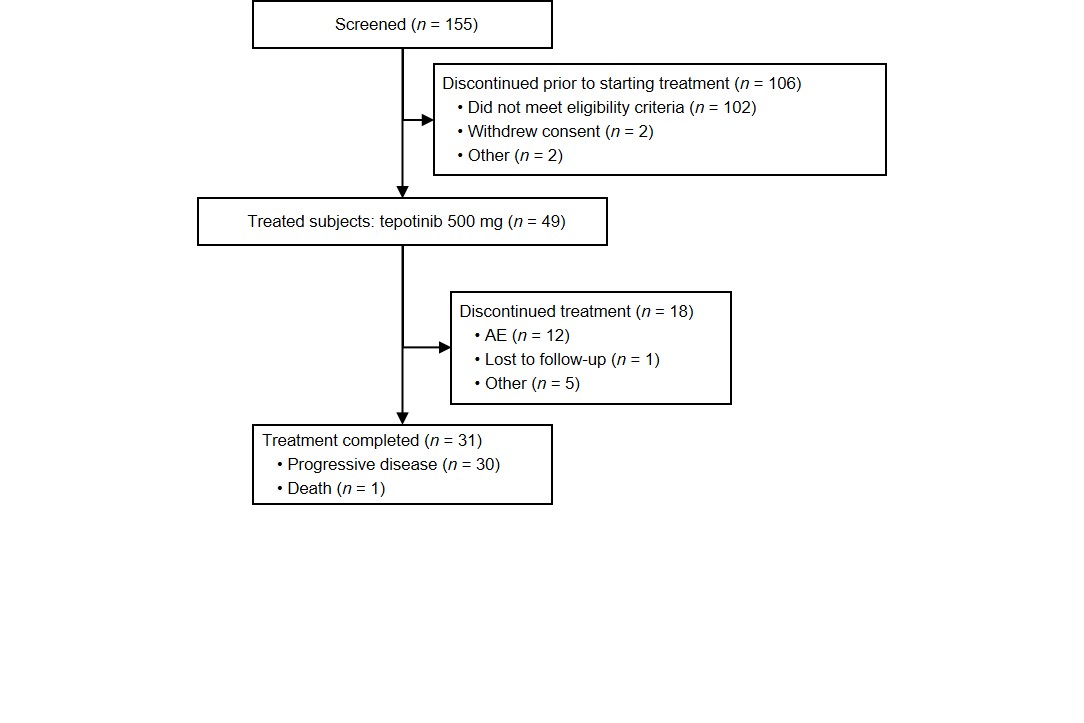
**

*AE* adverse event.

## **Supplementary Fig. 3.** Phase II: Kaplan–Meier curves for progression-free survival (investigator-assessed) (**a, c, e**) and overall survival (**b, d, f**), according to HBV/HCV status (**a, b**), MET IHC (**c, d**), or MET amplification (**e, f**)

**a.**

**
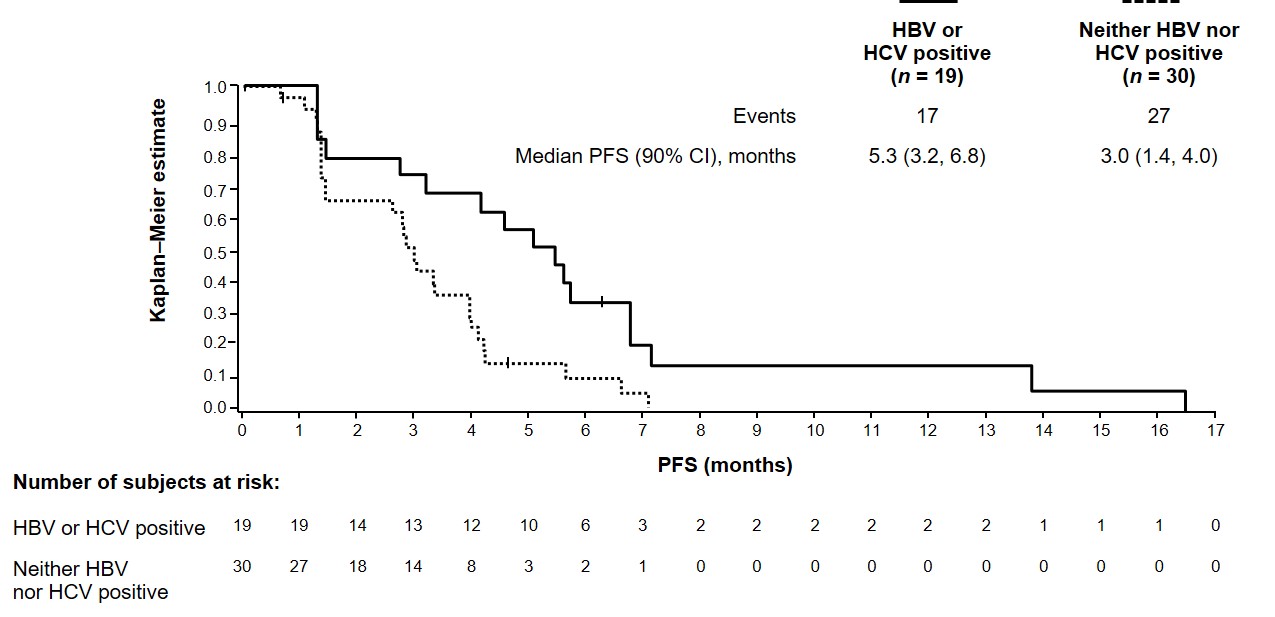
**

**b.**

**
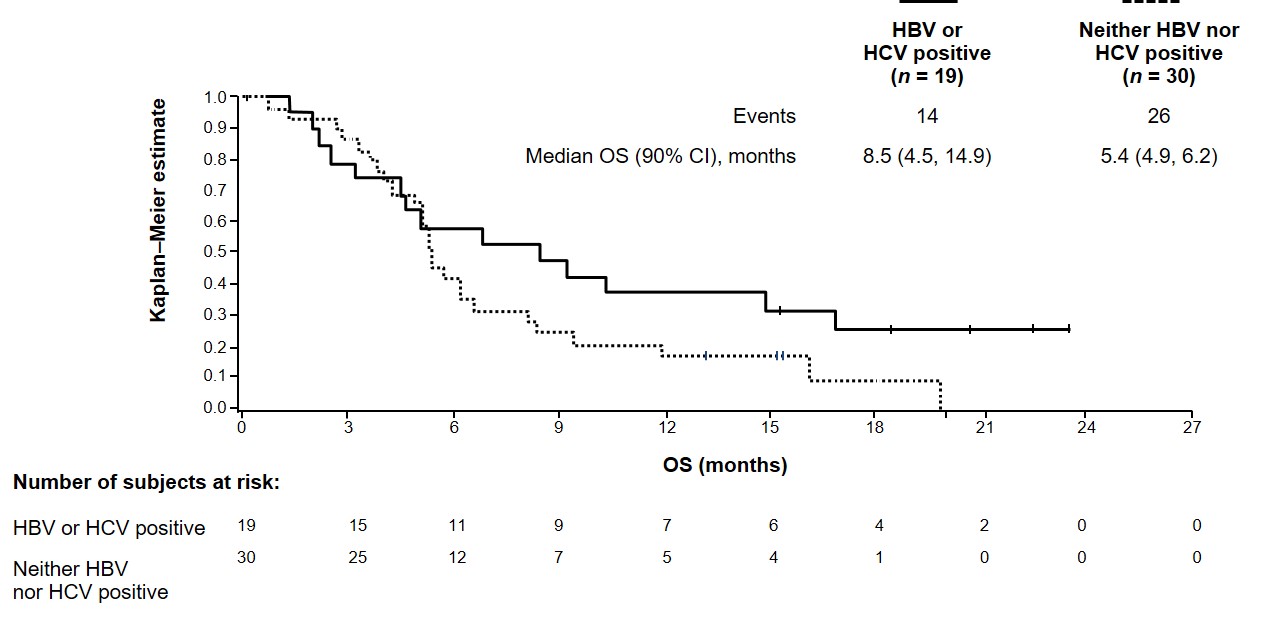
**

**c.**

**
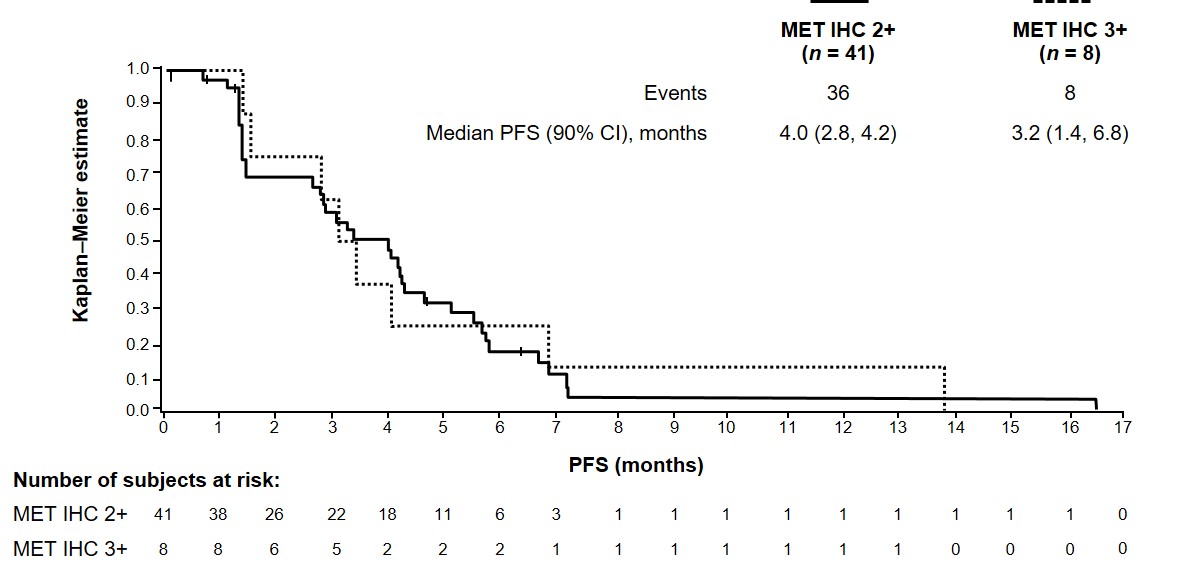
**

**d.**

**
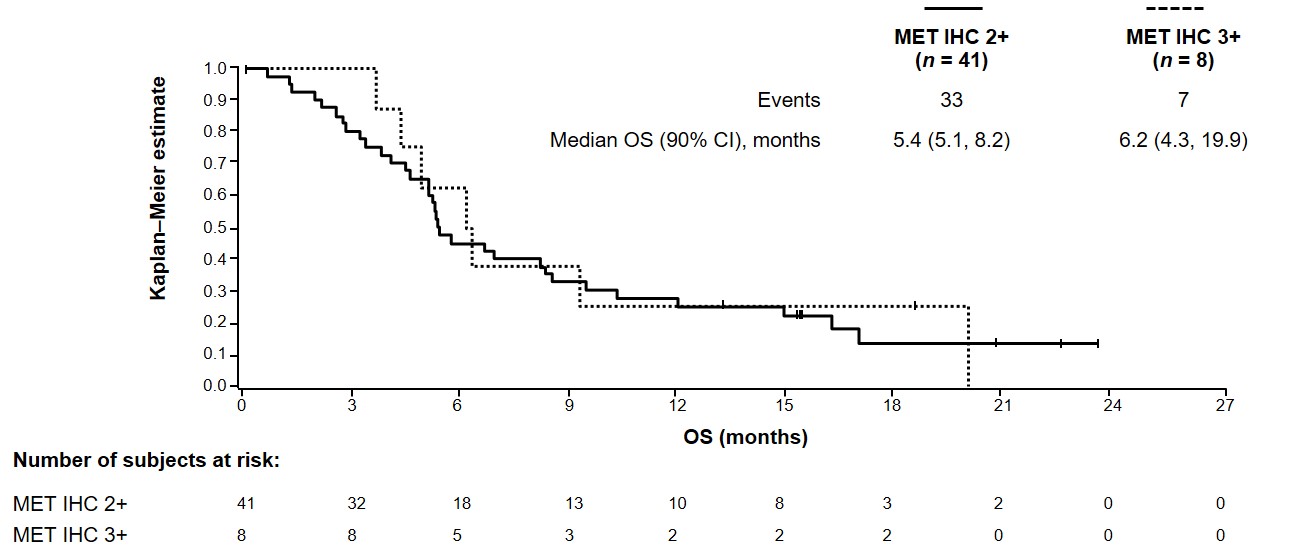
**

**e.**

**
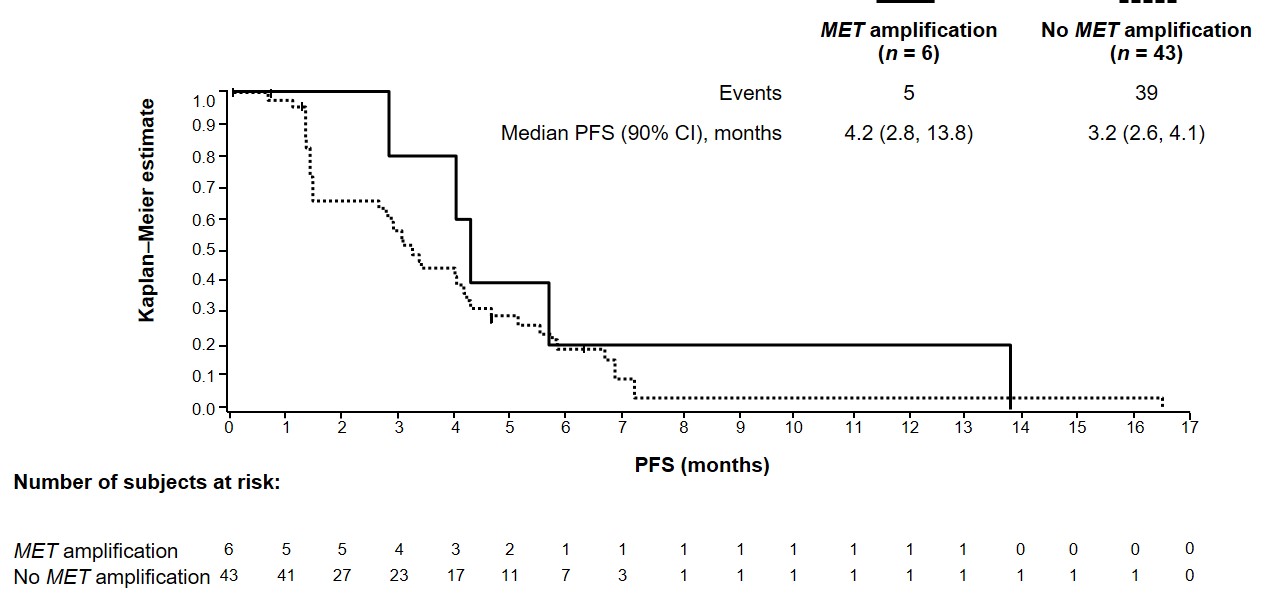
**

**f.**

**
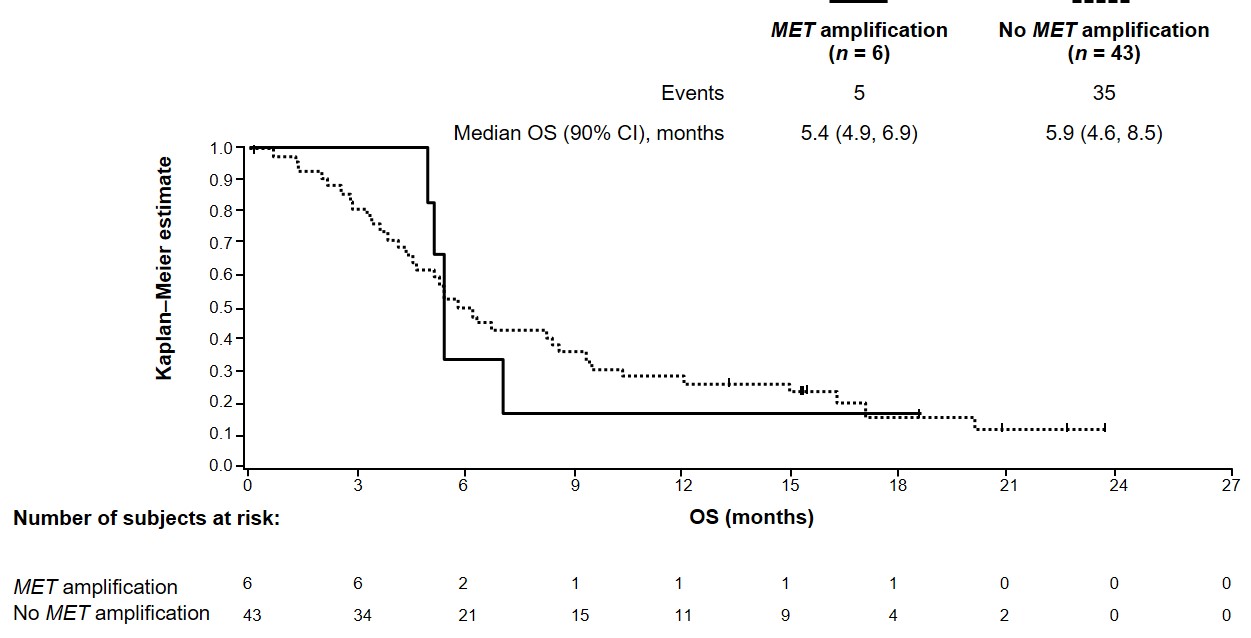
**

*CI* confidence interval, *HBV* hepatitis B virus, *HCV* hepatitis C virus, *IHC* immunohistochemistry, *OS* overall survival, *PFS* progression-free survival.
